# Eating Patterns, Body Mass Index, and Food Deserts: Does It Matter Where We Live?

**DOI:** 10.5888/pcd12.150352

**Published:** 2015-09-03

**Authors:** Samuel F. Posner

One of the great pleasures of being the Editor in Chief of *Preventing Chronic Disease: Public Health Research, Practice, and Policy* (*PCD*) is to read the papers submitted by the next generation of public health professionals for the annual *PCD* Student Contest. This year was no exception. We received 59 papers on a range of critical public health topics that used novel analytic methods. In collaboration with members of the Editorial Board, it is my pleasure to announce that Nelly Mejia at the Pardee RAND Graduate School has won the 2015 *PCD* Student Contest. In this paper, Mejia and colleagues describe their analysis of the association between living in a food desert and eating fruits and vegetables (1). Understanding the influence of food deserts on public health is critical to designing, implementing, and evaluating the impact of policy and environmental changes to improve access to nutritious foods.

Much of the published literature has documented both the prevalence of food deserts and disparities in access to nutritious foods (2–5). In the past several years, there has been a substantial effort to place farmers markers in areas considered to be food deserts (4–6). These interventions have documented some success; however, there are a number remaining challenges. Access to healthier foods is necessary but not sufficient to improve nutrition and mitigate the short-term and long-term health effects of suboptimal nutritional intake. Interventions must also address issues of preparation of these healthier foods for consumption. Access and purchasing behavior are first steps in changing dietary intake.

Mejia and colleagues conducted an analysis of the association of living in a food desert on consumption of fruits and vegetables in Los Angeles County. Their findings question current evidence about outlet density, access, healthy behaviors, and health status. In this setting, they found no consistent evidence that consumption of healthier foods or body mass index (BMI) were associated with density of food outlets commonly reported to carry less healthful foods. The finding was especially convincing because of the number of comparisons and the lack of any statistical associations detected. The findings of this study do not definitively answer the question of the relationship between access, intake, and BMI. No study will have sufficient sample size to “prove” there is no association.

There are contextual variables that potentially mediate the findings here that may not be relevant in other settings. Los Angeles County has a population of approximately 10 million; the county is highly diverse economically, socially, and culturally (7). Although Los Angeles County is known for urban and suburban sprawl, parts of the northeast portion of the county are remote and sparsely populated. This diversity makes the findings of this study relevant to one of the most populous regions of the country but may not easily translate to other settings. Nonetheless, the findings of this study are important to the field, because they point to the importance of contextual factors that can mediate the associations that we are most interested in studying. Health status and health behaviors do not often have simple and direct relationships.

Mejia and colleagues demonstrate the changes in measurement and analytic techniques that can lead to different conclusions. In this case the results were surprisingly consistent given the number of permutations and analytic approaches. Geographic boundaries can either highlight or mask important findings. In this study, there was little variation in the findings regardless of size or shape of unit of geographic analysis. Looking at both more proximal outcomes of consumption and distal measures of BMI also produced little difference in the conclusions. Rarely in chronic disease prevention are interventions straightforward and rarely do they consistently impact health behavior and health status in a multitude of settings.

This work represents some of the best of the up-and-coming generation of public health professionals. *PCD* is proud to be able to contribute to the career development of the next generation of researchers and practitioners. The changes faced in chronic disease prevention and health promotion are complex and not always as they appear at first glance. As with any study, this one does not provide a definitive answer on how food deserts are related to consumption patterns and BMI. Objectively conducting analysis and using data to understand the burden of disease, especially when the results may not be consistent with the current understanding, is how science progresses. Mejia and colleagues have done exactly that and helped advance the understanding of the relationship between access to healthier foods and intermediate outcome measures of diet and nutrition.
