# Erratum, Vol. 12, September 3 Release

**DOI:** 10.5888/pcd12.150352e

**Published:** 2015-09-24

In the article “Eating Patterns, Body Mass Index, and Food Deserts: Does It Matter Where We Live?”, there was an error in reference 1. The correct reference is: Mejia N, Lightstone AS, Basurto-Davila R, Morales DM, Sturm R. Neighborhood food environment, diet, and obesity among Los Angeles County adults, 2011. Prev Chronic Dis 2015;12:150078. DOI: http://dx.doi.org/10.5888/pcd12.150078.

The corrections were made to our website September 4, 2015, and appear online at http://www.cdc.gov/pcd/issues/2015/15_0352.htm. We regret any confusion or inconvenience this error may have caused.

